# Pulmonary artery compliance is associated with mortality but lacks predictive utility

**DOI:** 10.1016/j.jhlto.2026.100539

**Published:** 2026-03-17

**Authors:** Jacqueline T. DesJardin, Matthew Broerman, Melissa Saul, Darren Haskett, Seyed M. Nouraie, Marc A. Simon

**Affiliations:** a Department of Medicine; Division of Cardiology; University of California San Francisco, San Francisco, CA; b Department of Medicine; Division of Pulmonary, Allergy, Critical Care, and Sleep Medicine, Pulmonary Translational Research Core; University of Pittsburgh, Pittsburgh, PA; c Department of Surgery; Division of Cardiac Surgery; University of Maryland, Baltimore, MD

**Keywords:** Pulmonary artery compliance, Pulmonary hypertension, Predictive analytics, Pulmonary vascular resistance, Hemodynamics

## Abstract

**Background:**

Pulmonary artery compliance has physiologic appeal in pulmonary hypertension, but its clinical relevance remains in question.

**Methods:**

Adults undergoing right heart catheterization 2005–2019 at University of Pittsburgh Medical Center were included. For association analysis, Cox proportional hazards and linear tail-restricted cubic spline models were used to analyze the relationship between pulmonary artery compliance and mortality. The adjusted hazard for mortality in low (*<* 3 mL/mmHg) versus high (>3 mL/mmHg) pulmonary artery compliance were analyzed in multiple hemodynamic subgroups. For prediction analysis, resampling-based model comparisons were used to develop training (for model fitting) and testing (for model performance evaluation) datasets. Full models including hemodynamic and clinical variables were compared to “handicapped” models in which a variable of interest (i.e., pulmonary artery compliance) was removed using the Harrell’s Concordance index and Bayesian methods.

**Results:**

Among 12,866 patients with 68,444 person-years of follow-up, there were 4946 deaths. Mortality risk progressively increased with declining pulmonary artery compliance. Low pulmonary artery compliance was associated with a 54% increased hazard of death (HR 1.54; 95%CI 1.45 – 1.61, p<0.001) in adjusted Cox models. However, removal of pulmonary artery compliance from full predictive Cox models did not substantially diminish predictive performance for mortality on hold-out testing data (mean Harrell’s Concordance Index 0.703 versus 0.704, p=0.541).

**Conclusions:**

Although low pulmonary artery compliance is associated with increased mortality, inclusion of pulmonary artery compliance in multivariate predictive models does not improve mortality prediction. Pulmonary artery compliance does not substantially contribute to predictive performance for mortality.

## Introduction

In pulmonary hypertension (PH), quantification of the right ventricular afterload is essential for classifying disease and determining candidacy for pulmonary vasodilator therapy. The pulmonary vascular resistance (PVR) measured on right heart catheterization (RHC) is typically used to assess right ventricular afterload, with PVR values above 2 Wood units considered to be abnormal.^1^

Pulmonary artery compliance (PAC) is another measure of right ventricular afterload which is calculated by dividing the stroke volume by the pulmonary artery pulse pressure (SV/PP). While PVR measures steady state afterload, PAC measures pulsatile afterload, or the energy necessary to overcome elevated pressure during ventricular systole.^2^ Proponents of PAC note that the pulmonary circulation is highly pulsatile and PAC contributes approximately 25% to the right ventricular afterload, and 1.2- to 18-fold more to the right ventricular stroke work index than PVR.^2^, ^3^, ^4^ Both resting and exercise PAC have been proposed to be more sensitive markers of pulmonary vascular disease than PVR and have been associated with prognosis in several subpopulations of PH.^2^, ^5^, ^6^, ^7^, ^8^ Nonetheless, prior literature on PAC has several limitations and the clinical relevance of this hemodynamic parameter remains in question. First, PAC normal values remain unclear. The European Society of Cardiology and European Respiratory Society (ESC/ERS) defines a normal PAC as > 2.3 mL/mmHg; however, recent data suggest that the appropriate cutoff may be higher.^9^ Additionally, despite its physiologic appeal, it is unclear if PAC provides useful clinical information over the well-established hemodynamic variables already in use, namely PVR and mean pulmonary artery pressure (mPAP).

A recent study by Wang et al., 2023 provides strong evidence that PAC is significantly associated with mortality in two large RHC databases – U.S. Veteran’s Affairs and Vanderbilt University.^9^ Adjusted all-cause mortality was found to progressively increase with declining PAC, and a PAC < 3 mL/mmHg was significantly associated with mortality in multiple hemodynamic subgroups (i.e., high pulmonary artery wedge pressure [PAWP], low PAWP, high PVR, low PVR). While these data provide strong evidence for an association between PAC and mortality in patients with PH, they do not prove that PAC predicts mortality in manner which is practical for clinical use. Although often conflated, association and prediction are different statistical questions which necessitate different statistical techniques and “a significant statistical association is insufficient to establish a claim of prediction.”^10^ Therefore, the clinical question remains: does consideration of a patient’s PAC improve a clinician’s ability to *predict* mortality, above and beyond the other readily available clinical and hemodynamic variables?

In this study we used a large RHC database from the University of Pittsburgh Medical Center (UPMC) to assess 1) the association between PAC and mortality, and 2) the added utility of PAC for prediction of mortality. For the association analysis, we use comparable methods to Wang et al., 2023. For the prediction analysis, we employ a strategy of validation widespread in the machine learning community but rare in clinical predictive models: resampling-based model comparison according to predictive accuracy.

## Materials and methods

### Data source and study population

The Clinical Data Repository Enhanced Right Heart Catheterization Database is a single-institution database from the UPMC which includes catheterization, clinical, and administrative data. The development of this database has previously been described.^11^ This study was approved by the Institutional Review Board at the University of Pittsburgh.

**Figure 1:**
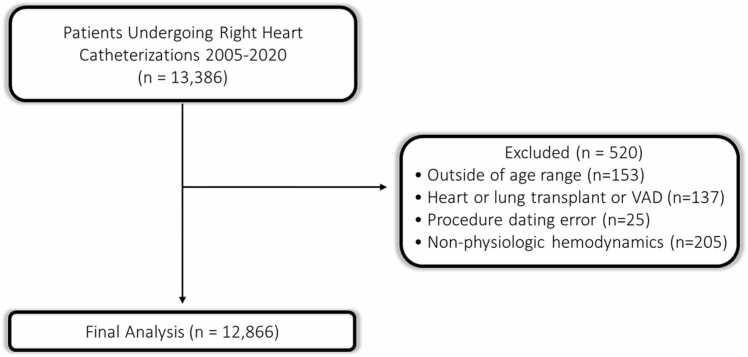
Consort Diagram.

This cohort included all patients who underwent RHC at UPMC facilities between January 2005 and December 2019 with follow-up data until November 2020. Only index RHC was included for each patient. Exclusion criteria were age under 18 or over 90 years, history of heart transplant or ventricular assistance device, procedure dating error (procedure date recorded after follow-up date), or non-physiologic hemodynamics (Figure 1). Non-physiologic hemodynamics were considered to be: (1) cardiac output (CO) <0.5 or >15 L/min; (2) mean pulmonary artery pressure (mPAP) <5 or >80 mm Hg; (3) diastolic pulmonary artery pressure (dPAP) <0 or >70 mmHg; (4) systolic pulmonary artery pressure (sPAP) <7 or >130 mmHg; (5) pulmonary artery wedge pressure (PAWP) <0 or >60 mm Hg; (6) heart rate (HR) <10 or >200 bpm, as has been used in prior studies.^12^

### Hemodynamic definitions and calculations

Right heart catheterizations were performed at rest using 5 to 7 French balloon-tipped catheters by experienced advanced heart failure or interventional cardiologists. Standard pressure measurements included the right atrial pressure (RAP), sPAP, dPAP, mPAP, PAWP, and HR. Pressure measurements were obtained at end-expiration. The CO was determined using the thermodilution technique in 95% of cases, with use of the indirect Fick method in 5% of cases when thermodilution was not available. Thermodilution cardiac output was generally recorded as the mean of at least 3 measurements. Calculated hemodynamic variables were derived by the following equations:

Pulmonary pulse pressure (PP) = sPAP – dPAP

Transpulmonary Gradient (TPG) = mPAP – PAWP

Pulmonary vascular resistance (PVR) = TPG / CO

Stroke volume (SV) = CO / HR

Pulmonary arterial compliance (PAC) = SV / PP

PH was defined as mPAP > 20 mmHg. Subgroups of PH, including pre-capillary PH (mPAP > 20 mmHg, PAWP ≤ 15 mmHg, PVR > 2 Wood units), isolated post-capillary PH (mPAP > 20 mmHg, PAWP > 15 mmHg, PVR ≤ 2 Wood units, [IpcPH]), combined post- and pre-capillary PH (mPAP > 20 mmHg, PAWP > 15 mmHg, PVR > 2 Wood units, [CpcPH]), and undetermined PH were defined by hemodynamic criteria based on the 2022 ESC/ERS Guidelines.^1^ Undetermined PH were patient who met criterial for PH based on mPA > 20 mmHg, but whose combination of hemodynamic variables was not consistent with any subgroup defined by the 2022 ESC/ERS Guidelines.^1^ For example, an individual with a mPA >20 mmHg, PAWP ≤ 15 mmHg, and a PVR < 2 Wood units would be considered “undetermined.”

### Clinical covariates

Demographic data including age, gender, and race were extracted. Age was defined by the patient’s age at time of the RHC procedure. Covariates were extracted based on administrative and charge data, as described in our prior publication.^11^ The Charlson Comorbidity Index (CCI), which takes into account multiple medical comorbidities and has been validated to predict mortality risk in multiple populations across multiple conditions, was calculated for each patient.^13^, ^14^ The CCI is a weighted scoring system that assigns points for conditions spanning multiple organ systems which are relevant to all World Health Organization (WHO) PH groups. Nonetheless, given pre-existing data on the association between body mass index (BMI), atrial fibrillation, obstructive sleep apnea, and hypertension with adverse outcomes, especially in PH due to left heart disease, information on these comorbidities was additionally collected. We felt this was crucial given it is estimated that >70% of patients with PH have PH due to left heart disease.^1^

### Exposure and outcome

The exposure under consideration was PAC, as calculated by stroke volume divided by pulmonary pulse pressure. The study outcome was all-cause mortality following RHC, determined as previously reported.^11^

### Statistical analysis of association

Cox proportional hazards models were used to analyze time to event data for all-cause mortality. Time 0 was considered to be the date of the RHC. Covariates in the model included age, gender, race, BMI, CCI, atrial fibrillation, obstructive sleep apnea, and hypertension. Cox models were assessed for proportional hazards violation. A linear tail-restricted cubic spline model with 4 knots was constructed to describe the relationship between PAC, as a continuous variable, and the hazard ratio for mortality. The unadjusted relationship was modeled first, and then the model was adjusted for covariates, using the most frequent or median value for these variables as the reference. A PAC of 3 mL/mmHg was considered to be the reference in order to remain consistent with prior publications.^9^ To allow for direct comparison to Wang et al., 2023, the adjusted hazard ratios for mortality when PAC was dichotomized to *<* or > 3 mL/mmHg were also calculated for the following subgroups: 1) PAWP < 15 mmHg, 2) PAWP > 15 mmHg, 3) PVR < 2.2 Wood units, and 4) PVR > 2.2 Wood units. A sensitivity analysis excluding those without PH was performed.

### Statistical analysis of prediction

**Figure 2:**
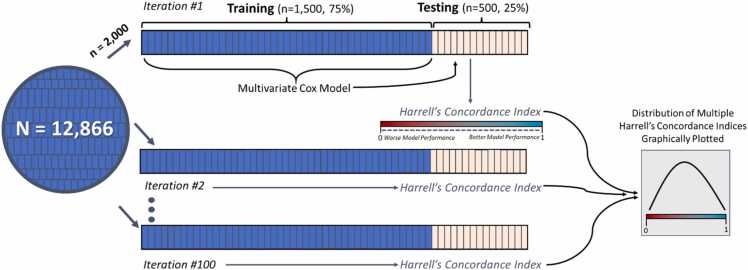
Schematic representation of Monte Carlo Cross Validation Methods with derivation and plotting of Harrell’s Concordance Indices.

To determine predictive performance of models for mortality, we used Monte Carlo cross-validation methods (Figure 2). Two-thousand subjects were sampled from the full database at random 100 times. Within each sample of 2000 subjects, 1500 subjects were used to train and 500 were used to test. For each set, a Cox model was fit to the training data of 1500 subjects. The model’s performance was then evaluated on the hold-out test set of 500 subjects. Predictive power in the test set was evaluated with the Harrell’s Concordance index, and the distribution of 100 instances of this performance metric was plotted graphically (Figure 2). The initial Cox model included age, gender, race, BSA, CCI, atrial fibrillation, OSA, hypertension, mPAP, and PAC. The same process was then repeated for alternative versions in which the Cox model was “handicapped” by removing a variable of interest (age, mPAP, or PAC). The mPAP was chosen as the comparator for PAC because it is directly measured, readily available, and is already used clinically. Differences in model performance between the full model and handicapped models were represented graphically and quantitated using analysis of variance (ANOVA) to evaluate the change in distribution of Harrell’s Concordance indices. A handicapped model which demonstrated a significant decline in the average Harrell’s Concordance Indices (compared to the full model) indicated that the removed variable contributed significantly to predictive performance. In addition to using ANOVA to evaluate the distributions of Harrell’s Concordance indexes, we compared model performances using Bayesian ANOVA alternative methods and computed the posterior region of practical equivalence (ROPE). Subgroup analysis was performed based on PH subtype (no PH, pre-capillary PH, IpcPH, or CpcPH) and PVR sub-group (<2, 2–3, 3–5, and >5 WU). A sensitivity analysis excluding those without PH was performed.

## RESULTS

The final study included 12,866 patients who underwent RHC at UPMC between 2005 and 2019. The study sample was 55% male, 84% white, and had a median age of 64 years old (Table 1). The median PAC was 2.4 mL/mmHg (IQR 1.5 – 3.7) and CpcPH was the most common form of PH, affecting 29% of patients.

**Table 1:** Baseline patient characteristics at time of right heart catheterization

	Total (N=12,866)
Age, years, Median (IQR)	64 (55, 73)
Sex, n (%N)	
Male	7076 (55%)
Female	5840 (45%)
Race, n (%N)	
White	10,821 (84%)
Black or African American	1310 (10%)
Unknown	642 (5%)
Other	93 (1%)
Body Mass Index, kg/m^2^, Median (IQR)	28 (24, 34)
Charlson Comorbidity Index, Median (IQR)	2 (1, 4)
Comorbidities, n (%N)	
Atrial Fibrillation	3356 (26%)
Hypertension	8768 (68%)
Obstructive Sleep Apnea	2129 (17%)
Hemodynamics, Median (IQR)	
Heart rate	75 (64, 87)
Mean pulmonary artery pressure [mPAP], mmHg	28 (21, 38)
Systolic pulmonary artery pressure [sPAP], mmHg	43 (33, 58)
Diastolic pulmonary artery pressure [dPAP], mmHg	16 (11, 23)
Pulmonary artery wedge pressure [PAWP], mmHg	15 (10, 22)
Pulmonary vascular resistance [PVR], Wood units	2.4 (1.5, 3.9)
Pulmonary artery compliance [PAC], mL/mmHg	2.4 (1.5, 3.7)
Cardiac output [CO], L/min	4.9 (3.7, 6.2)
Pulmonary Hypertension Type, n (%N)	
Combined post- and pre-capillary pulmonary hypertension [CpcPH]	3781 (29%)
Pre-capillary pulmonary hypertension	3063 (24%)
No pulmonary hypertension	2927 (23%)
Isolated post-capillary pulmonary hypertension [IpcPH]	2198 (17%)
Undetermined pulmonary hypertension	897 (7%)

CpcPH: mPAP > 20 mmHg, PAWP > 15 mmHg, PVR > 2 Wood units; Pre-capillary: mPAP > 20 mmHg, PAWP ≤ 15 mmHg, PVR > 2 Wood units; IpcPH: mPAP > 20 mmHg, PAWP > 15 mmHg, PVR ≤ 2 Wood units

### Association of PAC and mortality

There was a total of 68,444 person-years of follow-up. At a median follow-up of 4.4 (IQR 1.4, 8.5) years there were 4946 deaths. The risk of mortality progressively increased with declining PAC values on both unadjusted (Figure 3a) and adjusted analyses (Figure 3b) below a PAC of ∼5 mL/mmHg. Low PAC (<3 mL/mmHg) was associated with a 54% increased hazard of death (HR 1.54; 95%CI 1.45 – 1.61, p<0.001) in the adjusted Cox models. As has been previously shown,^9^ PAC < 3 mL/mmHg was independently associated with increased risk of mortality among subgroups of patients with low PAWP, high PAWP, low PVR, or high PVR (Table 2), even when restricting analysis to only those with PH (Supplemental Table 1).

**Figure 3a:**
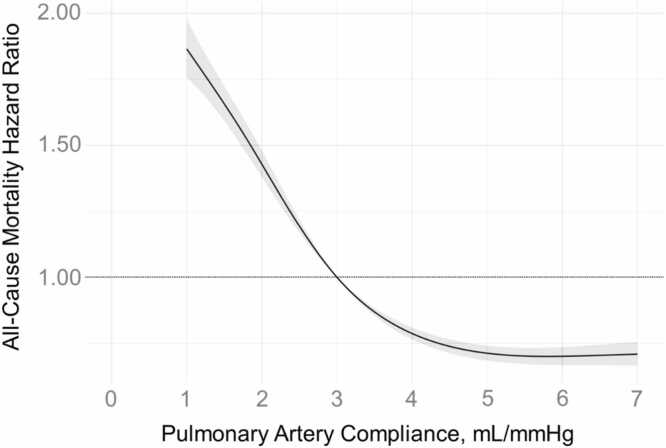
The unadjusted hazard ratio for mortality according to pulmonary artery compliance.

**Figure 3b:**
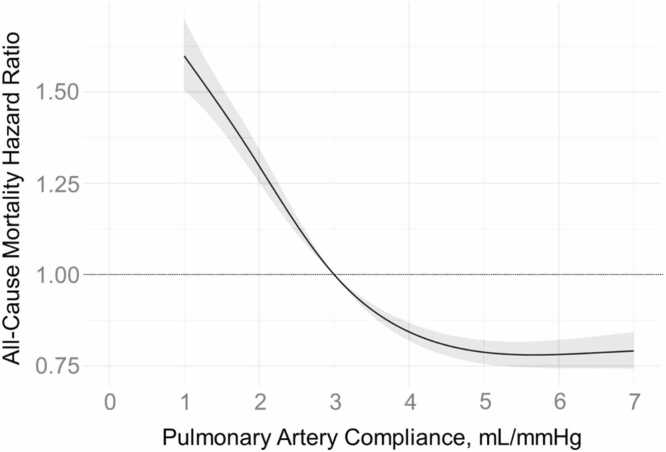
The adjusted hazard ratio for mortality according to pulmonary artery compliance. Adjusted model controlled for age, gender, race, body surface area, Charlston Comorbidity Score, atrial fibrillation, obstructive sleep apnea, and hypertension.

**Table 2:** Hazard ratios for mortality by hemodynamic subgroups

	Adjusted All-Cause Mortality		
Variable	N	HR (95% CI)	*P*
Low PAC, PAC ≤ 3 mL/mmHg	8121	Ref	-
High PAC, PAC > 3 mL/mmHg	4745	0.65 (0.62 – 0.69)	<0.001
Low PAC & Low PAWP, PAC < 3 mL/mmHg + PAWP ≤ 15 mmHg	3554	Ref	-
High PAC & Low PAWP, PAC ≥ 3 mL/mmHg + PAWP ≤ 15 mmHg	3288	0.65 (0.65 – 0.70)	<0.001
Low PAC & High PAWP, PAC < 3 mL/mmHg + PAWP > 15 mmHg	4567	Ref	-
High PAC & High PAWP, PAC ≥ 3 mL/mmHg + PAWP > 15 mmHg	1457	0.76 (0.70 – 0.82)	<0.001
Low PAC & High PVR, PAC < 3 mL/mmHg + PVR > 2.2 WU	6186	Ref	-
High PAC & High PVR, PAC ≥ 3 mL/mmHg + PVR > 2.2 WU	878	0.72 (0.65 – 0.80)	<0.001
Low PAC & Low PVR, PAC < 3 mL/mmHg + PVR ≤ 2.2 WU	1935	Ref	-
High PAC & Low PVR, PAC ≥ 3 mL/mmHg + PVR ≥ 2.2 WU	3867	0.75 (0.69 – 0.81)	<0.001

Covariates controlled for: age, gender, race, body surface index, Charlston Comorbidity Index, atrial fibrillation, obstructive sleep apnea, and hypertension

### Prediction of mortality by PAC

**Figure 4:**
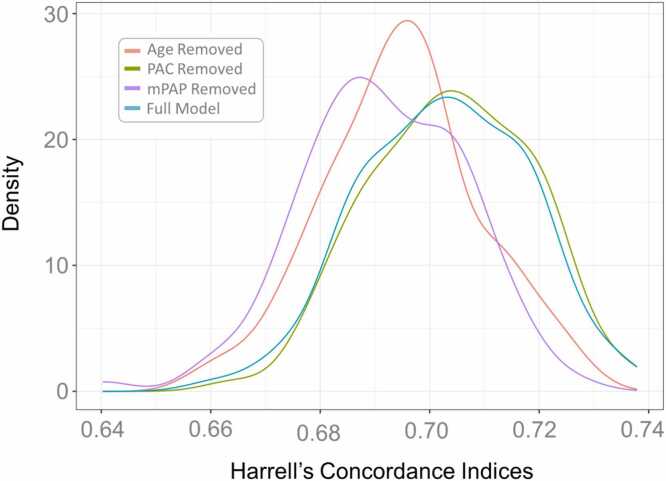
Impact of Predictor Variable Removal on Model Performance: Distribution of Harrell’s Concordance Indices for Full Model and Each Handicapped Model. Full model included age, gender, race, body surface area, Charlson Comorbidity Index, atrial fibrillation, obstructive sleep apnea, hypertension, mean pulmonary artery pressure (mPAP), and pulmonary artery compliance (PAC).

**Figure 5:**
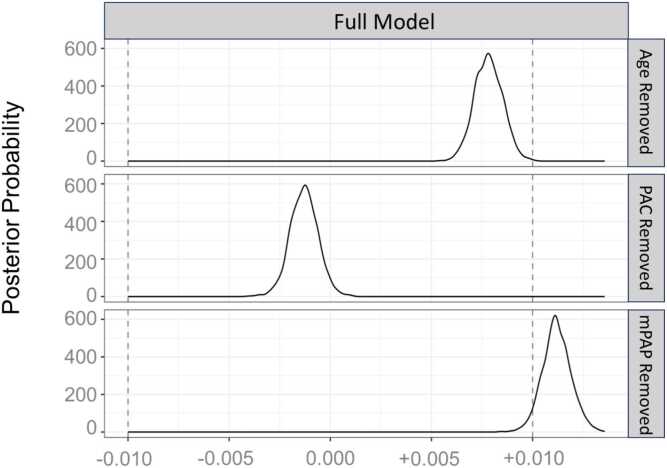
Impact of Predictor Variable Removal on Model Performance: Bayesian Analysis of Variance with Posterior Region of Practical Equivalence (ROPE). Full model included age, gender, race, body surface area, Charlson Comorbidity Index, atrial fibrillation, obstructive sleep apnea, hypertension, mean pulmonary artery pressure (mPAP), and pulmonary artery compliance (PAC). Posterior probability distributions of the difference in model performance after removing individual variables (Age, PAC, and mPAP) from the full model. The region of practical equivalence (ROPE) is set at an effect size of 0.01 (dashed lines).

**Figure 6:**
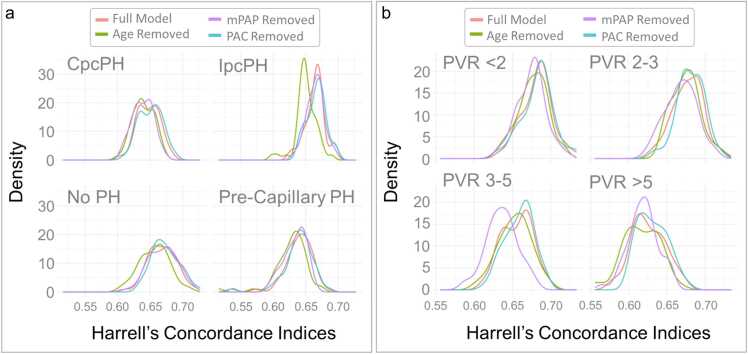
Impact of Predictor Variable Removal on Model Performance Among Subgroups. **a**. Pulmonary Hypertension Subgroups. **b** Pulmonary Vascular Resistance Subgroups. CpcPH = combined post- and pre-capillary pulmonary hypertension; IpcPH = isolated post-capillary pulmonary hypertension; PH = pulmonary hypertension; PVR = pulmonary vascular resistance (in Woods units). Full model included age, gender, race, body surface area, Charlson Comorbidity Index, atrial fibrillation, obstructive sleep apnea, hypertension, mean pulmonary artery pressure (mPAP), and pulmonary artery compliance (PAC).

For the fully-adjusted Cox model, which included the hemodynamic variables mPAP and PAC, the median Harrell’s Concordance index on 100 partitions of testing data was 0.703 + 0.001 (95%CI 0.700 – 0.705; Figure 4). After removal of PAC, the predictive performance did not change significantly and the distribution of Harrell’s Concordance indices was similar to the full model (Figure 4**;** difference of mean 0.001, p=0.531), indicating that PAC was not substantially contributing to predictive performance in a model that also contains mPAP and clinical variables. Comparatively, when either mPAP or age were removed from the full model, predictive performance significantly dropped (−0.008, −0.011, p<0.001 respectively; Figure 4), indicating that these variables are important for mortality prediction. On sensitivity analysis excluding those without PH, the findings were similar with mPAP and age but not PAC significantly contributing to model performance, although the absolute importance of mPAP was attenuated compared to the main analysis (Supplemental Figure 1). Using the Baysian ANOVA approach with an effect size set at 0.01 (ROPE = 0.01), removal of either age or PAC did not significantly impact model performance, as their posterior distributions remain within the ROPE (Figure 5). However, removing mPAP leads to a practically meaningful difference, suggesting its importance in predicting mortality (Figure 5). Overall, the results suggest that mPAP significantly contributes to the prediction of mortality in multivariate models whereas PAC does not. PAC also did not significantly predict mortality on subgroup analysis by PH type or PVR subgroup **(**Figure 6**)**.

## Discussion

We found that PAC is significantly associated with mortality in a large database of patients receiving RHC at the University of Pittsburgh Medical Center. Below 5 mL/mmHg, PAC has a linear inverse relationship with all-cause mortality, consistent with findings in prior publications.^9^ Although PAC is significantly associated with mortality in Cox models, consideration of PAC does not improve accuracy for prediction of all-cause mortality in models which already include readily available clinical and hemodynamic variables.

The finding that PAC is independently associated with mortality but does not improve prediction of mortality may at first seem contradictory, but in actuality underscores the important statistical and practical distinction between measures of association and measures of prediction. Association and prediction are frequently conflated in medical literature, and the term “prediction” is often incorrectly used to refer to a statistical association or correlation, including in prior literature on PAC.^6^, ^8^, ^15^, ^16^ At a minimum, predictive statistics require a derivation cohort for initial model fit and an independent validation cohort to test model performance.^10^ Machine learning techniques, such as those used in this study, have improved predictive statistics by employing cross-validation which efficiently develops derivation and validation cohorts to robustly test predictive performance. The statistical tests required to establish prediction are different than those which prove association, and so it should not be surprising that many variables which are statistically associated are not necessarily useful for predicting outcomes. Therefore, results deemed significant on the basis P-values from measures of association alone may misguide clinical practice when clinicians are actually interested in predicting adverse outcomes.

There is a growing PH and heart failure literature in which new, calculated hemodynamic metrics are being developed and associated with outcomes in an attempt to better characterize right ventricular afterload and function; examples include PAC, diastolic pressure gradient, trans-pulmonary pressure gradient, total pulmonary vascular resistance, pulmonary artery pulsatility index, RAP to PAWP ratio, right ventricular stroke work index, and cardiac power output.^17^, ^18^ Many of these metrics make physiologic sense and may be useful in specific circumstances, but as increasingly complex calculations are derived, we must remember that all rely on the same basic measured data: heart rate and blood pressure; right atrial, pulmonary artery, and wedge pressures; and cardiac output or index. Before incorporating these new derived metrics into clinical practice, we need to ensure that they represent a substantial improvement over readily available, familiar, and interpretable parameters.

In the case of PAC, PAC and PVR share mathematical components and are designed to measure different aspects of the same thing: the right ventricular afterload. The variables are inherently linked via the resistance-compliance time (RC) such that RC = PAC x PVR.^19^, ^20^ In health and disease, the PAC and PVR demonstrate a close inverse hyperbolic relationship, although it has been described that the RC time shortens slightly in certain PH subgroups,^21^, ^22^ as well as with higher left atrial filling pressures,^20^ older age,^23^ and higher heart rate.^24^ Nonetheless, given the relatively tight relationship between PAC and PVR, one wonders how much additive value PAC could provide to predict outcomes beyond PVR, it’s mathematical cousin. The common practice of dichotomizing PAC and then assessing the association of PAC with outcomes in PVR subgroups (as we demonstrate in Table 2 of this paper) can result in the misleading interpretation that PAC is associated with mortality independent of the PVR. In reality, the PVR and PAC are both continuous variables, and interval changes in PVR and/or PAC above and below their arbitrarily defined cut points may provide prognostic value that is being disregarded when the variable is dichotomized.^25^

Despite the physiologic appeal of the PAC, its clinical relevance remains in question and it has not been widely adopted by clinicians.^26^ Although some studies, such as Wang et al., 2023 and our study, have demonstrated a strong association between PAC and survival in PH, others have not.^9^ Perhaps the strongest case for considering PAC is among patients with idiopathic, hereditary, or drug-induced pulmonary arterial hypertension (PAH) who fulfill acute vasoreactivity response criteria. Gerhardt and colleagues recently demonstrated that a large increase in the PAC during acute vasoreactivity testing was associated with a favorable long-term response to calcium channel blocker therapy, suggesting the PAC may be useful to consider in this subpopulation of PAH patients.^16^ However, no prior studies on PAC (including Gerhardt et al., 2024) employed the predictive analytic methods necessary to draw predictive conclusions. In fact, prior research has shown that even though PAC is associated with survival in PAH, it does not add prognostic value over the validated risk prediction tools developed using predictive analytic methods (e.g., REVEAL 2.0 risk calculator).^22^ The fundamental question on the minds of clinicians is whether or not spending the extra time to calculate and interpret PAC will provide any clinically meaningful and actionable information beyond readily available hemodynamics. To our knowledge, this is the first study to rigorously distinguish between association and prediction when evaluating PAC's clinical utility. Based on the results of our study, we question whether meaningful risk-prediction can be derived from consideration of PAC when clinicians already have access to other hemodynamic and clinical data. The large database was a strength of this study, allowing for us to use machine-learning resampling techniques which require large sample sizes. Our use of resampling-based model comparison to assess predictive accuracy was a strength and demonstrates a methodological advance compared to prior studies. However, several limitations should be noted. The population was derived from a single institution with a relatively racially homogenous (84% white) population. This study is limited by data quality confines and unavailable or missing data which is inevitable in any large retrospective observational study. The hemodynamic values were recorded as a part of standard clinic care and are therefore subject to waveform artifacts that can impair data quality and measurement accuracy. We have previously shown that dPAP is systematically underestimated due to motion artifact in retrospective non-adjudicated databases such as this UPMC database.^11^ While this may lead to underestimation of the true PAC, our results likely have external validity when comparing to other institutions that use unadjudicated measurements in routine clinical care. Analyzing PAC as a continuous (rather than dichotomized) variable was a strength of this study and would have mitigated some of the risks of this bias. More detailed clinical information, such as medications or therapies (before or after RHC) as well as WHO clinical PH group was not available. Hemodynamic indices alone are not sufficient to clinically classify patients with PH, and so although we looked at hemodynamic subgroups (i.e., pre-capillary PH) we were unable to analyze the utility of PAC in clinical subgroups (i.e., WHO Group 1 PAH). The UPMC database does not include exercise hemodynamics, and so the potential utility of exercise PAC cannot be examined in the current study. Furthermore, we only examined the relationship between PAC and all-cause mortality. Other hemodynamic parameters, such as the Pulmonary Artery Pulsatility Index, may also benefit from a similar rigorous evaluation distinguishing association from prediction, which we did not address in this focused analysis. It is also possible that PAC is useful in predicting other outcomes besides mortality such as response to medical therapy or hospitalizations, which we could not assess in the present study. Further analysis examining the relationship between PAC and cause-specific mortality may find that PAC is predictive of certain types of mortality, such as cardiovascular-specific death. We did not find that PAC was useful in predicting mortality among various PH or PVR subgroups, such as patients with pre-capillary PH or low PVR. We studied the utility of PAC in all-comers to the cardiac catheterization laboratory; however, PAC may prove useful in certain subpopulations such as those with vasoreactive PAH.^16^

In a large single-institution database, PAC is independently associated with mortality but does not improve prediction of mortality beyond routinely considered hemodynamic and clinical variables. This study serves as a reminder that measures of association do not imply predication, the latter of which requires derivation and validation cohorts to develop and test predictive models. Our methodological approach (resampling-based model comparison with derivation and validation cohorts) represents the appropriate statistical framework for answering prediction questions. By demonstrating how to properly evaluate predictive utility, we hope to provide a methodological template for the field and future studies on novel hemodynamic metrics.

## FINANCIAL DISCLOSURE STATEMENT

JTD and MAS note salary support from National Institutes of Health grant funding (JTD - T32HL007731, 5KL2TR001870–10; MAS - 1R01AG058659). No relevant financial conflicts of interest reported.

## Footnotes

Appendix A Supplemental data associated with this article can be found in the online version at doi:10.1016/j.jhlto.2026.100539.
