# Supplementary material for: Pulmonary artery compliance is associated with mortality but lacks predictive utility
Source: JHLT Open. 2026 Mar 17;12:100539. doi: 10.1016/j.jhlto.2026.100539 (PMC13100256; doi:10.1016/j.jhlto.2026.100539)
Supplement: Supplementary file 1 — Supplemental material [file mmc1.docx]

**Supplemental Figure 1.** Sensitivity Analysis excluding patients without PH – Impact of Predictor Variable Removal on Model Performance: Distribution of Harrell’s Concordance Indices for Full Model and Each Handicapped Model


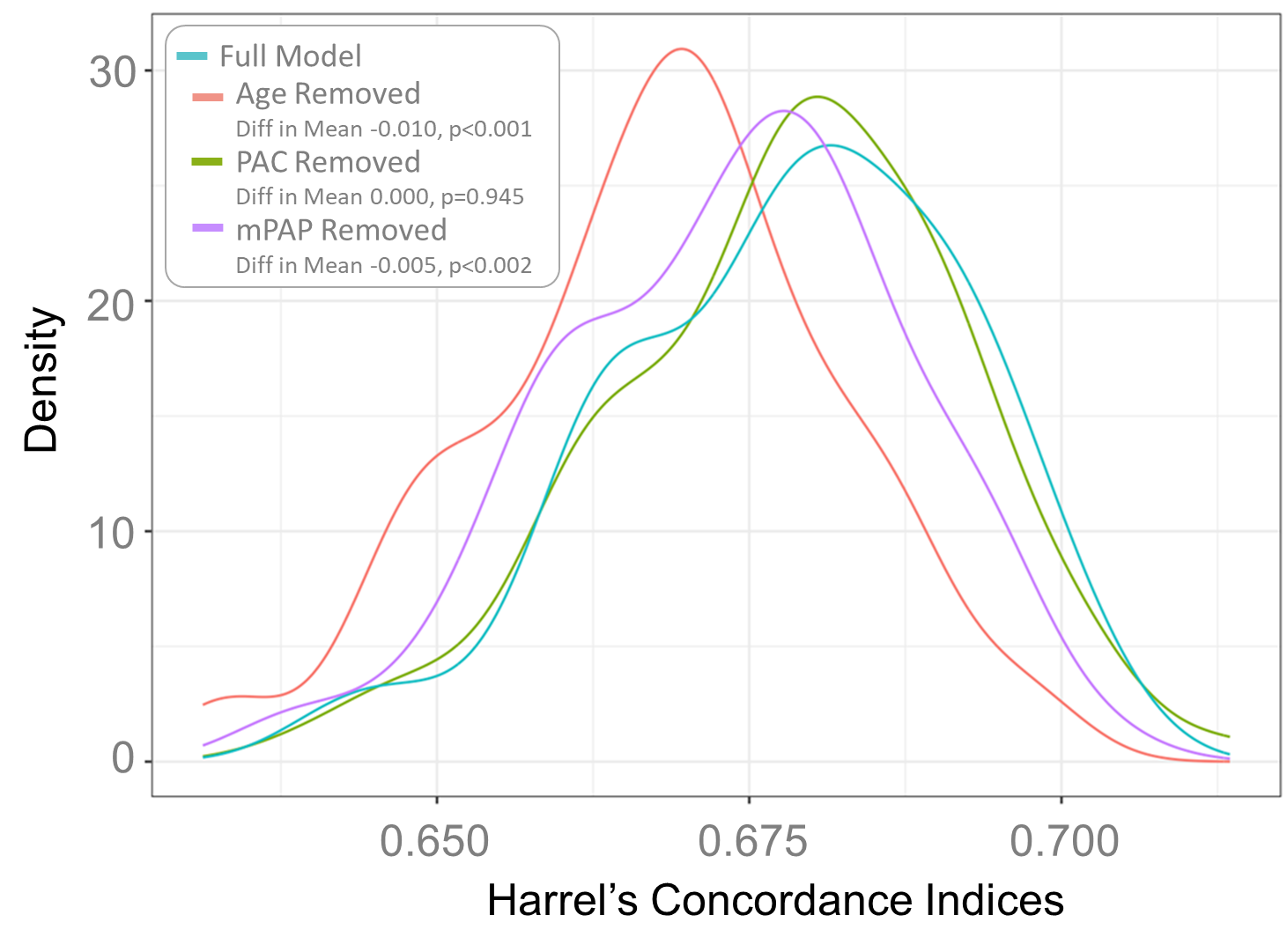


Full model included age, gender, race, body surface area, Charlson Comorbidity Index, atrial fibrillation, obstructive sleep apnea, hypertension, mean pulmonary artery pressure (mPAP), and pulmonary artery compliance (PAC).

**Supplemental Table 1**. Sensitivity Analysis excluding patients without pulmonary hypertension – hazard ratios for mortality by hemodynamic subgroup

|  | **Adjusted All-Cause Mortality** | | |
| --- | --- | --- | --- |
| **Variable** | **N** | **HR (95% CI)** | ***P*** |
| **Low PAC**, PAC < 3 mL/mmHg | 7,208 | Ref | - |
| **High PAC**, PAC > 3 mL/mmHg | 2,731 | 0.70 (0.66 – 0.75) | <0.001 |
| **Low PAC & Low PAWP**, PAC < 3 mL/mmHg + PAWP < 15 mmHg | 2,656 | Ref | - |
| **High PAC & Low PAWP**, PAC > 3 mL/mmHg + PAWP < 15 mmHg | 1,304 | 0.65 (0.59 – 0.72) | <0.001 |
| **Low PAC & High PAWP**, PAC < 3 mL/mmHg + PAWP > 15 mmHg | 4,552 | Ref | - |
| **High PAC & High PAWP**, PAC > 3 mL/mmHg + PAWP > 15 mmHg | 1,427 | 0.76 (0.70 – 0.82) | <0.001 |
| **Low PAC & High PVR**, PAC < 3 mL/mmHg + PVR > 2.2 WU | 5,633 | Ref | - |
| **High PAC & High PVR**, PAC > 3 mL/mmHg + PVR > 2.2 WU | 568 | 0.74 (0.67 – 0.83) | <0.001 |
| **Low PAC & Low PVR**, PAC < 3 mL/mmHg + PVR < 2.2 WU | 1,575 | Ref | - |
| **High PAC & Low PVR**, PAC > 3 mL/mmHg + PVR < 2.2 WU | 2,073 | 0.76 (0.70 – 0.84) | <0.001 |

Analysis included only patients with pulmonary hypertension (n=9,939); there was a median follow-up of 3.8 years (IQR 1.2, 7.8) with 4,018 deaths. Covariates controlled for: age, gender, race, body surface index, Charlston Comorbidity Index, atrial fibrillation, obstructive sleep apnea, and hypertension
